# Quality and Implementation of Diabetic Care at a Free Clinic

**DOI:** 10.51894/001c.30026

**Published:** 2022-02-24

**Authors:** Noumi Chowdhury, Mark Trottier, Ghufraan Akram

**Affiliations:** 1 Michigan State University College of Human Medicine; 2 Office of Research Michigan State University College of Human Medicine; 3 HUDA Clinic

**Keywords:** Type 2 Diabetes, Diabetes care, Quality of care, Quality Improvement

## Abstract

**INTRODUCTION:**

Although typically receiving little government funding, free clinics help ensure access to affordable quality health care to the medically underserved. Established in 2004, the authors’ metropolitan Detroit Health Unit on Davison Avenue (HUDA) Clinic delivers primary care and specialized services to uninsured populations. The authors compared proportionate changes in A1c levels compared to prior national averages to evaluate the significance of care a free diabetes clinic can provide to uninsured populations.

**METHODS:**

Retrospective data from 2017-2019 were reviewed of adult patients who have been diagnosed with Type 2 Diabetes. From HUDA Clinic medical records, data were collected concerning patient demographics, insurance status, pregnancy, major comorbidities and several factors related to diabetes standards of care.

**RESULTS:**

There were a total of 2,231 patient visits to HUDA Clinic in 2019, of which 125 patients (5.6%) received care for their Type 2 diabetes. Forty (32%) clinic patients who had a visit in 2019 had an HbA1c <7.0 and 29 (23%) had an HbA1c > 9.5. This is comparable to the 2020 National Diabetes Statistics Report in which approximately 50% (n = 15.6 million) of Americans had an HbA1c < 7.0 and 14% (n = 5.1 million) had an HbA1c > 9.5.

**CONCLUSIONS:**

Huda Clinic’s diabetes care percentages were quite comparable to state and national data and CDC parameters, although these comparative results need to be considered in terms of the authors’ smaller sample size. These overall results indicate that health care providers can meet major recommended diabetic care at inner-city free clinics in metropolitan communities. Future provider and patient study studies regarding free clinic care patterns are clearly required to identify gaps in healthcare access and formulate and test specific strategies to improve diabetes-related outcomes.

## INTRODUCTION

According to the National Association of Community Health Centers (NACHC),^1^ community health centers were developed during the civil rights era as a part of the “War on Poverty” to mitigate health outcome disparities and empower communities through federal funds to generate neighborhood clinics. These community health centers integrated federal, regulatory, and state affairs with policy research and advocacy to improve health outcomes and preventative measures to benefit the medically underserved and uninsured by performing data collection and analysis.^1^

Free clinics, according to The National Association of Free & Charitable Clinics (NAFC),^2^ generally serve a similar function as community-based clinics helping ensure the medically underserved access to affordable health care services. However, free (and charitable) clinics typically receive little to no government funding and are funded via different means depending on the clinic. According to the Michigan Department of Human Health and Services 2018 Free Clinic Report,^3^ a total of $250,000 was allocated to be equally distributed among thirty-nine organization (i.e., approx. $6,400 per clinic) during that year providing “safety net” care to approximately 34,044 uninsured patients.

Founded in 2004, The Health Unit on Davison Avenue (HUDA) Clinic in the metropolitan Detroit area has aimed to bring primary care and specialized services to uninsured populations. The clinic has maintained their own primary care services including health assessments, acute and chronic illness screening, management and education, laboratory services. free medications, etc.^4^ The clinic has also developed academic partnerships with the Michigan State University Colleges of Human Medicine and College of Osteopathic Medicine.

Key Type 2 Diabetes (T2D) care services for HUDA clinic patients include screening and treatments (with a full physical exam including a monofilament and blood glucose test), individual and group diabetes management education, provision of diabetes-related medications, an ongoing urban garden and having an annual “Project Happy Feet” program providing free podiatric care. Clinic providers were assigned to provide each patient with necessary laboratory work, patient education, medication, and glucometer supplies as needed at no cost. ^4^

At the time of this study, the authors had identified few recent studies regarding diabetes care outcomes in free clinics. Many of these studies were older than ten years. The most relevant study found was the 2009 Ryskina et al.^5^ study in which an East Harlem Health Outreach Partnership in diabetes management outcomes was assessed using glycosylated hemoglobin level and blood pressure control along with other indicators.

The authors also found limited Hemoglobin A1C average data from statewide reports. However, national data from the Centers for Disease Control and Prevention (CDC) was extensive in providing national averages for diabetic management parameters. In terms diabetes management goals, the CDC listed 2020 National Diabetics Statistics Report^6^ updated goals for A1c, blood pressure, cholesterol and smoker status. The recommended goals were a HbA1c <7.0, BP<140/90, Chol <130, and nonsmoker status. The CDC also listed less stringent goals with HbA1c <8.0, BP <140/90, LDL <160, and nonsmoker status.^6^

## Purpose of Study

The purpose of this pilot study was to compare HUDA Clinic T2D care patterns to recommended standards of care, determine percentage of T2D patients with well-controlled diabetes, and compare these results to previous published outcomes.

## METHODS

The authors used a retrospective descriptive chart review design. Before data collection, the study was IRB-approved as exempt. The study population was comprised of HUDA clinic adults who were 18 and older who have been previously or newly diagnosed with T2D. This excludes females with maternal diabetes and adults with Type 1 Diabetes or pre-diabetes (i.e., metabolic syndrome).

From the medical records at HUDA Clinic, data was collected by the first author (NC) regarding patient demographics, insurance status, pregnancy, comorbidities and several factors related to diabetes standard of care. Diabetes standard of care-related factors included:

- HbA1C testing: poor control (HbA1C > 9.5), good control (HbA1C < 7)
- Lipid panel monitoring: Lipids controlled at LDL <130 mg/dL
- Nephropathy monitoring
- Blood pressure: controlled at <140/90, blood pressure controlled at <130/80 mm. Hg.
- Retinopathy screen
- Foot exams

The first author (NC) collected information concerning patients’ Medicaid/Medicare status and medication availability/use as additional factors affecting T2D care to compare the patient population to other populations as well. The first author (NC) accessed patient medical records for extraction of data.

Data extraction only occurred using limited access, password-protected computers. A correlation tool was employed which designated each subject with a study identification number to be associated with the medical record number for each clinic patient. A separate data collection sheet contained all de-identified health and demographic data collected for each patient, along with the patient assigned study identification number. No identifying information was included in the data collection sheet. Data were stored in password protected Microsoft Excel files and saved on password-protected computers.

The first author (NC) calculated percentages of study patients who returned and received recommended T2D screening tests by the clinic. Any changes in individual standard of care-related factors were reviewed for comparison.

## RESULTS

According to the 2018 HUDA Clinic annual report, the clinic provided 1,246 office visits with approximately 810 (65%) of those regular patients falling below the poverty line. The majority of the patients HUDA Clinic provided care to in 2018 self-identified as being from a minority background as well as being currently unemployed (Table 1).

In 2019, there were a total of 2,231 patient visits to HUDA Clinic. Out of 125 patients (5.6%) came to visit regarding their T2D. Out of these 125 patients the average age among patients was 57 years old (SD = 11.0). Fifty (40%) of the patients were male while 75 (60%) were female. The average BMI was 30.6 (SD 8.0) which would classify average patients as “obese.” A subgroup of 33 (26.4%) patients had a current of former history of smoking. Furthermore, 96 (76.8%) patients had additional comorbidities such as hypertension, hyperlipidemia, hypercholesteremia, or hypertriglyceridemia (Table 2).

The average HbA1c was an 8.0 % (SD = 2.0), which is marked by a “good” degree of control and “good health risk” according to the Mayo Clinic and American Diabetes Association.^7^ The average blood glucose was at 188 mg/dl, (SD = 58.0) which was in between an “Excellent” and “Good” degree of control and between a “low” and “good” health risk (Table 2).^7^

**Table 1.** HUDA Clinic 2018 Patient Statistics

**Race/Ethnicity**	Black	White	Asian	Middle-Eastern	Hispanic	Other
	55% N=687	9% N=105	22% N=275	6% N=80	2% N=22	6% N=77
**Job Status**	Full-time	Part-time	Retired	Unemployed	Student	Self-Employed
	23% N=286	10% N=124	8% N=99	49% N=610	5% N=62	6% N=74
**Poverty Status**	<100%	100-200%	>200%			
	65% N=809	29% N=361	6% N=74			

**Table 2.** HUDA Clinic 2019 Type 2 Diabetics Statistics

Total 18+ Type 2 Diabetes	Age Avg	Men	Women	BMI	Smokers	Additional Comorbidity	A1C Avg	Glucose	Cholesterol	HDL	LDL
125	57 (11)	40% N=50	60% N=75	30.6 (8)	26.4% N=33	76.8% N=96	8% (2)	185 (65)	173 (45)	46 (25)	99 (43)

## DISCUSSION

According to Mayo Clinic, in 2018,^7^ it was recommended for an individual to have at least two HbA1c tests drawn every year. During 2019, 56.7% of the Type 2 Diabetes HUDA Clinic patients had at least two HbA1c tests in comparison to 65.7% State of Michigan Department of Health and Human Services estimates (Table 3).^3^

**Table 3.** HUDA Clinic Comparison with State and National Data

	2 HbA1C Tests Obtained	HbA1C Good <7%	HbA1C Poor >9.5%	LDL <130 mg/dl	BP >140/90 mm Hg	BMI >40	Former Smoker	Current Smoker
HUDA Clinic	56.7% N=70	32% N=40	26% N=32	76.5% N=95	60.8% N=76	10.4% N=13	10.4% N=13	16% N=20
Michigan ^3^ (N=87,000)	65.7% N=57,159	--	--	44.1% N=38,367	53.5% N=46,545	--	--	20.3% N=17,661
National ^6^ (N=34.2 million)	--	50% N=17.1 million	14% N=5.8 million	60% N=20.5 million	56% N=19.1 million	61.3% N=20.9 million	36.4% N=12.4 million	36.6% N=12.5 million

National data collected for Type 2 diabetic management was organized in accordance to CDC parameters with an HbA1c of <7.0%. considered as good and HbA1c > 9.5%. considered as poor. According to the 2020 National Diabetes Statistics Report,^6^ 50% of Americans had an HbA1c < 7.0% and 14% had an HbA1c > 9.5%. This is in comparison to HUDA Clinic’s 32% with an HbA1c < 7% and 26% with an HbA1c > 9.5%. This left 42% of patient at the study clinic at an HbA1c between 7 and 9.5%.

HUDA Clinic also had high averages of patients with an LDL <130 mg/dl at 76.5% in comparison to the national average of 60%. The HUDA Clinic had lower averages of patients with a BMI > 40 at 10.4% versus the national average of 61.3%. The HUDA Clinic also had a lower average of patients with a history of smoking at 26.4% in comparison to 36.4% of the U.S. population (Table 3).

Since this study was based off the 2009 Ryskina, et. al.,^5^ HUDA clinic parameters of diabetic treatment and care of their T2D population was compared to Ryskina et al.^5^ and other studies. HUDA Clinic had a slight decrease in comparison’s to Ryskina et. al.^5^ in terms of individuals with an HbA1c <7.0%. However, HUDA clinic had a decreased number of individuals with an HbA1c >9.5% in comparison to the Ryskina et. al.^5^ study. HUDA Clinic also had the highest average of patients with an LDL <130 mg/dl. at 75%. In terms of retinopathy screens, HUDA Clinic was one of the lowest in terms of the number of patients to have received retinopathy screens and foot exams (Table 4).

**Table 4.** HUDA Clinic Comparison with Previous Studies

	HbA1C Good <7%	HbA1C Poor >9.5%	LDL <130 mg/dl	Retinopathy Screen	Foot Exam
HUDA Clinic (n=125)	32% N=40	26% N=32	77% N=96	27.2% N=34	3.2% N=4
EHHOP^5^ (n=147)	38% N=55	38% N=55	60% N=88	92% N=135	88% N=129
National Report^6^ (n=2677)	50.9% N=1362	63.8% N=1707	--	--	--
National Report (CDC)^8^ (n=34.1 million)	--	--	--	58.7% N=20 million	76.7% N=26.1 million
DHOP^9^ (n=189)	--	28% N=52	--	27% N=51	62% N=117
New York Medicaid^10^ (n=1.7 million)	--	37% N=629,000	--	56% N=952,000	73% N=1.2 million
National Medicaid^11^ (n=34.1 million)	--	48.7% N=16.6 million	--	51.4% N=17.5 million	--
National Commercial Health Plans^11^ (n=34.1 million)	--	29.6% N=10 million	--	--	--

Most patients that the HUDA Clinic serves are minority patients, with most patients also unemployed and below the poverty line. In comparison to national averages,^8,9,12^ the HUDA Clinic had a lower percentage of patients with an HbA1c >9.5% and a lower percentage of patients with a BMI >40 and patients with a history of smoking, including former smokers and current smokers. The study clinic also had a higher percentage of patients with an LDL <140. The Huda Clinic did, however, have a high average of patients with BP >140/90 mm. Hg., lower percentage of patients with an HbA1C <7.0%, and a slightly lower average of patients who receive Hb A1Cs within a year in comparison to the overall average across the state of Michigan and nationally.^3,10,13^

When compared to other studies HUDA Clinic demonstrated equivalent of somewhat better, HbA1C and LDL parameters. However, study clinic averages were also low with the percentage of patients that received a retinopathy screen and foot exam. Many of the parameters used to compare HUDA Clinic’s data to other established results were according to parameters of diabetic care set by the CDC and thus this review is a good display of how well they meet the CDC parameters, especially when these parameters are compared to other established results.

## Study Limitations

There were certainly several limitations to our study design. Updated data from other free clinic settings concerning patients with T2D was difficult to obtain, especially studies concerning specific HbA1C levels and other major diabetes care parameters. Our review was limited to 2019 eligible HUDA Clinic patients. In 2017, the HUDA Clinic had established an electronic health record (EHR), and providers may have still been adjusting to their newer documentation systems.

Future provider and patient study studies regarding free clinic T2D care patterns are clearly required to identify gaps in healthcare access and formulate and test strategies to improve diabetes-related outcomes.

## CONCLUSIONS

Based on these pilot results, similar diabetes care outcomes can be achieved at free inner-city clinics compared to more typical primary care settings. The authors readily acknowledge that future larger sample studies employing inferential statistics comparisons of T2D care outcomes from multiple sites are required to more fully investigate care in these complex primary care settings.

### Conflict of Interest

None.

### Submission Date

07/13/2021

### Acceptance Date

11/22/2021
